# Author Correction: Systematic experimental comparison of particle filtration efficiency test methods for commercial respirators and face masks

**DOI:** 10.1038/s41598-022-12766-5

**Published:** 2022-05-18

**Authors:** Joel C. Corbin, Greg J. Smallwood, Ian D. Leroux, Jalal Norooz Oliaee, Fengshan Liu, Timothy A. Sipkens, Richard G. Green, Nathan F. Murnaghan, Triantafllos Koukoulas, Prem Lobo

**Affiliations:** Metrology Research Centre, National Research Council Canada, Ottawa, ON K1A 0R6 Canada

Correction to: *Scientific Reports* 10.1038/s41598-021-01265-8, published online 09 November 2021

The original version of this Article contained an error.

The number and surface areas of respirators measured by Roberge et al.^43^ were incorrectly quoted. Roberge et al. measured nine (not 12) respirators.

As a result, in the Results and discussion section, under the subheading ‘Face Velocity’,

“For example, Roberge et al.^43^ reported the total inner-layer surface area of 12 N95 respirators as ranging from 108 to 205 cm^2^ (mean ± 2 standard deviations, 146 ± 26 cm^2^). The mean area would result in a mean face velocity of 7.3 ± 1.9 cm s^−1^ for the flow rate of the NIOSH method. However, this mean area is an overestimate; a more accurate calculation would subtract the area of the mask in contact with the wearer’s face, which does not contribute to filtration. If this region comprised 10% of the inner-layer area, it would increase the mean face velocity to 8.1 ± 2.0 cm s^−1^. In this section and in Fig. 5, we conservatively use a range of 5.4 to 10.1 cm s^−1^, encompassing both of the above estimates, when comparing the face velocities relevant to the NIOSH standard with the ASTM F2299/F2100 standard.”

now reads:

“For example, Roberge et al.^43^ reported the total inner-layer surface area of nine N95 respirators as ranging from 158 to 255 cm^2^ (mean ± 2 standard deviations, 197 ± 57 cm^2^). These values result in a mean calculated face velocity of 7.3 ± 2.0 cm/s for the flow rate of the NIOSH method. However, this mean area is an overestimate, a more accurate calculation would subtract the area of the mask in contact with the wearer's face, which does not contribute to filtration. If this region comprised 10% of the inner-layer area, it would increase the mean face velocity to 8.1 ± 2.2 cm/s. In Fig. 5, we approximate this range of mean and standard deviations as 5.5 to 10 cm/s, when comparing the face velocities relevant to the NIOSH standard with the ASTM F2299/F2100 standard.”

The original Article has been corrected.

